# Supplementary material for: GluA4 facilitates cerebellar expansion coding and enables associative memory formation
Source: eLife. 2021 Jul 5;10:e65152. doi: 10.7554/eLife.65152 (PMC8291978; doi:10.7554/eLife.65152)
Supplement: Supplementary file 1. [file elife-65152-supp1.docx]

| **Table 1. Statistics.** | | | | | | | | |
| --- | --- | --- | --- | --- | --- | --- | --- | --- |
| **Figure 7** | **Test** | **Independent Variable** | **Dependent variable** | **n (mice)** | **F Statistic** | **DF1** | **DF2** | **p value** |
| D | mixed anova | genotype | str. length FR | 9, 12 | 0.04 | 1 | 97 | 0.85 |
| E | mixed anova | genotype | str. length HR | 9, 12 | 0.53 | 1 | 248 | 0.47 |
| F | mixed anova | genotype | inst velocity FR | 9, 12 | 3.03 | 1 | 256 | 0.08 |
| G | mixed anova | genotype | inst velocity HR | 9, 12 | 0.79 | 1 | 248 | 0.37 |
| H | mixed anova | genotype | z (mm) FR | 9, 12 | 10.10 | 1 | 54 | <0.05 |
| I | mixed anova | genotype | z (mm) HR | 9, 12 | 4.18 | 1 | 41 | 0.05 |
| J | mixed anova | genotype | %front-hind phase | 9, 12 | 1.61 | 1 | 256 | 0.21 |
| K | mixed anova | genotype | %left-right phase | 9, 12 | 0.02 | 1 | 259 | 0.90 |
| **Figure 7–figure supplement 1** | **Test** | **Independent Variable (IV)** | **Dependent variable** | **n (mice)** | **F Statistic** | **DF1** | **DF2** | **p value** |
| A | t-test, ind. | genotype | speed | 9, 12 |  |  |  | 0.017 |
| A | mixed anova | genotype | cv speed front | 9, 12 | 0.75 | 1 | 259 | 0.39 |
| A | mixed anova | genotype | cv speed hind | 9, 12 | 1.69 | 1 | 248 | 0.19 |
| B | mixed anova | genotype | cv swing length front | 9, 12 | 0.75 | 1 | 256 | 0.39 |
| B | mixed anova | genotype | cv swing length hind | 9, 12 | 0.10 | 1 | 248 | 0.75 |
| B | mixed anova | genotype | cadence front | 9, 12 | 0.03 | 1 | 91 | 0.86 |
| C | mixed anova | genotype | cv cadence front | 9, 12 | 1.90 | 1 | 254 | 0.17 |
| C | mixed anova | genotype | cadence hind | 9, 12 | 0.34 | 1 | 248 | 0.56 |
| C | mixed anova | genotype | cv cadence hind | 9, 12 | 0.12 | 1 | 248 | 0.73 |
| C | mixed anova | genotype | stance duration front | 9, 12 | 0.07 | 1 | 259 | 0.80 |
| D | mixed anova | genotype | cv stance duration front | 9, 12 | 3.45 | 1 | 259 | 0.06 |
| D | mixed anova | genotype | stance duration hind | 9, 12 | 1.36 | 1 | 248 | 0.24 |
| D | mixed anova | genotype | cv stance duration hind | 9, 12 | 1.53 | 1 | 248 | 0.22 |
| D | mixed anova | genotype | duty factor front | 9, 12 | 3.31 | 1 | 259 | 0.07 |
| E | mixed anova | genotype | cv duty factor front | 9, 12 | 0.01 | 1 | 259 | 0.93 |
| E | mixed anova | genotype | duty factor hind | 9, 12 | 0.20 | 1 | 248 | 0.66 |
| E | mixed anova | genotype | cv duty factor hind | 9, 12 | 0.01 | 1 | 248 | 0.92 |
| E | mixed anova | genotype | inst velocity front | 9, 12 | 3.03 | 1 | 256 | 0.08 |
| F | mixed anova | genotype | cv inst velocity front | 9, 12 | 0.09 | 1 | 259 | 0.77 |
| F | mixed anova | genotype | cv inst velocity hind | 9, 12 | 1.28 | 1 | 247 | 0.26 |
| G | mixed anova | genotype | cv z excursion front | 9, 12 | 0.02 | 1 | 259 | 0.89 |
| G | mixed anova | genotype | cv z excursion hind | 9, 12 | 7.17 | 1 | 88 | 0.01 |
| G | mixed anova | genotype | y excursion front | 9, 12 | 0.87 | 1 | 48 | 0.36 |
| H | mixed anova | genotype | y excursion hind | 9, 12 | 1.60 | 1 | 58 | 0.21 |
| H | mixed anova | genotype | base of support | 9, 12 | 0.29 | 1 | 56 | 0.59 |
| H | mixed anova | genotype | %double support front | 9, 12 | 2.40 | 1 | 259 | 0.08 |
| H | mixed anova | genotype | %double support hind | 9, 12 | 0.77 | 1 | 255 | 0.38 |
| I | mixed anova | genotype | cv front-hind phase | 9, 12 | 0.98 | 1 | 258 | 0.32 |
| I | mixed anova | genotype | cv left-right phase | 9, 12 | 1.37 | 1 | 258 | 0.24 |
| J | mixed anova | genotype | %3 paw supports | 9, 12 | 0.84 | 1 | 259 | 0.36 |
| J | mixed anova | genotype | %2 paw support others | 9, 12 | 3.87 | 1 | 258 | 0.07 |
| J | mixed anova | genotype | %2 front paw supports | 9, 12 | 0.35 | 1 | 259 | 0.55 |
| J | mixed anova | genotype | body y excursion | 9, 12 | 3.76 | 1 | 56 | 0.06 |
| K | mixed anova | genotype | tail y amplitude | 9, 12 | 0.76 | 1 | 24 | 0.39 |
| K | mixed anova | genotype | tail phase | 9, 12 | 40.40 | 1 | 79 | <0.01 |
| K | mixed anova | genotype | tail z amplitude | 9, 12 | 0.09 | 1 | 248 | 0.77 |
| K | mixed anova | genotype | nose y amplitude | 9, 12 | 8.61 | 1 | 259 | <0.01 |
| **Figure 7–figure supplement 3** | **Test** | **Independent Variable (IV)** | **Dependent variable** | **n (mice)** | **F Statistic** | **DF1** | **DF2** | **p value** |
| A | mixed anova | genotype | str. length FR | 9, 12 | 0.27 | 1 | 55.24 | 0.61 |
| B | mixed anova | genotype | str. length HR | 9, 12 | 3.36 | 1 | 69.52 | 0.07 |
| C | mixed anova | genotype | inst velocity FR | 9, 12 | 0.19 | 1 | 240.00 | 0.67 |
| D | mixed anova | genotype | inst velocity HR | 9, 12 | 0.85 | 1 | 227.00 | 0.36 |
| E | mixed anova | genotype | z (mm) FR | 9, 12 | 1.22 | 1 | 40.88 | 0.28 |
| F | mixed anova | genotype | z (mm) HR | 9, 12 | 1.47 | 1 | 46.28 | 0.23 |
| G | mixed anova | genotype | %front-hind phase | 9, 12 | 0.01 | 1 | 240.00 | 0.94 |
| H | mixed anova | genotype | %left-right phase | 9, 12 | 0.03 | 1 | 240.00 | 0.86 |
