# Supplementary material for: GluA4 facilitates cerebellar expansion coding and enables associative memory formation
Source: eLife. 2021 Jul 5;10:e65152. doi: 10.7554/eLife.65152 (PMC8291978; doi:10.7554/eLife.65152)
Supplement: Supplementary file 2. [file elife-65152-supp2.docx]

| **Table 2. GC IAF Model Parameters.** | | | | |
| --- | --- | --- | --- | --- |
| **Parameter** | **Unit** | **Value WT** | **Value KO** | **Source** |
| **Simulation and cell parameters** | | | | |
| Soma capacitance | pF | 4.30 | 4.30 | measured |
| G_leak_ | nS | 0.366 | 0.333 | Adjusted to reproduce GC rheobase (Figure S2B–C) |
| G_GABA_ | nS | 0.160 | 0 | Figure 2G |
| E_leak_ | mV | −110 | −110 | Adjusted to reproduce GC resting membrane potential |
| E_GABA_ | mV | −65 | −65 | calculated |
| E_AMPA_ | mV | 0 | 0 | calculated |
| E_NMDA_ | mV | 0 | 0 | calculated |
| AP threshold | mV | −45 | −45 | Figure 2 |
| AP threshold slope | mV | 2 | 2 | Brette and Gerstner 2005 |
| AP peak | mV | 32 | 32 | Figure 2 |
| AP reset | mV | −75 | −75 | Figure 2 |
| AP refractory time | ms | 1.5 | 1.5 | Mitchell and Silver 2003 |
| W τ | ms | 75 | 75 | Figure 2 |
| W a | nS | 0 | 0 | Figure 2 |
| W b | pA | 0.35 | 0.35 | Figure 2 |
|  |  |  |  |  |
| **Synaptic conductance** | | | | |
| G_AMPA_ direct_1 | nS | 1.4 | 0.196 | Figure 1C |
| G_AMPA_ direct_2 | nS | 0.55 | 0.077 | Figure 1C |
| τ_rise_ AMPA direct | ms | 0.33 | 0.45 | Rothman et al. 2009 / Figure 1C |
| τ_decay_ AMPA direct_1 | ms | 0.5 | 0.9 | Figure 1C |
| τ_decay_ AMPA direct_2 | ms | 4.0 | 7.2 | Figure 1C |
| G_AMPA_ spillover_1 | nS | 0.1 | 0.014 | Figure 1C |
| G_AMPA_ spillover _2 | nS | 0.25 | 0.035 | Figure 1C |
| G_AMPA_ spillover _3 | nS | 0.15 | 0.021 | Figure 1C |
| τ_rise_ AMPA spillover | ms | 0.9 | 1.1 | Figure 1C |
| τ_decay_ AMPA spillover_1 | ms | 0.8 | 1.44 | Figure 1C |
| τ_decay_ AMPA spillover_2 | ms | 4.0 | 7.2 | Figure 1C |
| τ_decay_ AMPA spillover_3 | ms | 30 | 54 | Figure 1C |
| G_NMDA_ direct | nS | 0.3 | 0.45 | Figure 5B |
| G_NMDA_ spillover | nS | 0.14 | 0.21 | Figure 5B |
| τ_rise_ NMDA | ms | 2.5 | 2.5 | Figure 5B |
| τ_decay_ NMDA direct | ms | 30 | 30 | Figure 5B |
| τ_decay_ NMDA spillover | ms | 70 | 70 | Figure 5B |
|  |  |  |  |  |
| **Short-term plasticity** | | | | |
| AMPA direct R inf |  | 1 | 1 | Figure 5C |
| AMPA direct R min |  | 0 | 0 | Figure 5C |
| AMPA direct τ R | ms | 85 | 57 | Figure 5C |
| AMPA direct P inf |  | 0.45 | 0.4 | Figure 5C |
| AMPA direct P min |  | inf | inf | Figure 5C |
| AMPA direct τ P | ms | −1 | −1 | Figure 5C |
| AMPA direct P scale |  | 0 | 0 | Figure 5C |
| AMPA spillover τ R | ms | 25 | 17 | Figure 5C |
| NMDA direct R inf |  | 1 | 1 | Figure 5C |
| NMDA direct R min |  | 0 | 0 | Figure 5C |
| NMDA direct τ R | ms | 50 | 50 | Figure 5C |
| NMDA direct P inf |  | 0.45 | 0.45 | Figure 5C |
| NMDA direct P min |  | inf | inf | Figure 5C |
| NMDA direct τ P | ms | 15 | 15 | Figure 5C |
| NMDA P scale | ms | 0.2 | 0.2 | Figure 5C |
