# Supplementary material for: GluA4 facilitates cerebellar expansion coding and enables associative memory formation
Source: eLife. 2021 Jul 5;10:e65152. doi: 10.7554/eLife.65152 (PMC8291978; doi:10.7554/eLife.65152)
Supplement: Supplementary file 3. [file elife-65152-supp3.docx]

| **Table 3. GC electrophysiological parameters (Figure 2)** | | | | | |
| --- | --- | --- | --- | --- | --- |
| **Genotype** | **Condition** | **N** | **Membrane resistance (GΩ)** | **Membrane capacitance (pF)** | **Series resistance (MΩ)** |
| WT | low chloride | 37 | 1.3 ± 0.1 | 3.9 ± 0.2 | 32.2 ± 2.1 |
| GluA4-KO | low chloride | 47 | 1.5 ± 0.2 | 3.6 ± 0.2 | 26.7 ± 1.5 |
| WT | high chloride | 8 | 1.7 ± 0.2 | 3.5 ± 0.6 | 21.7 ± 3.1 |
| GluA4-KO | high chloride | 7 | 2.0 ± 0.6 | 3.5 ± 0.6 | 21.1 ± 5.2 |
